# Clinical effectiveness and patient-reported outcomes of endovenous ablation and surgical stripping in varicose vein management: a systematic review

**DOI:** 10.1186/s12893-025-03269-x

**Published:** 2025-12-13

**Authors:** En Qing Lim, Aaron Jun Ket Lim, Aongus Twomey

**Affiliations:** 1 University of Dundee, Dundee, Scotland United Kingdom; 2 Department of Surgery, Cork University Hospital, Wilton, Co. Cork Ireland; 3 Universiti Teknologi Malaysia, Johor Bahru, Malaysia; 4 Department of Surgery, Mercy University Hospital, Grenville Place, Cork, T12 WE28 Ireland

**Keywords:** Varicose veins, Endovenous ablation, Radiofrequency Ablation (RFA), Endovenous Laser Ablation (EVLA), Mechanochemical Ablation (MOCA)

## Abstract

**Background/aims:**

Varicose veins affect 17% of females and 15% of males globally, significantly impairing quality of life. Endovenous ablation techniques, including thermal methods such as Endovenous Laser Ablation (EVLA) and Radiofrequency Ablation (RFA), as well as non-thermal approaches like Mechanochemical Ablation (MOCA) and cyanoacrylate adhesive (NBCA), offer minimally invasive alternatives to traditional surgery. This systematic review and meta-analysis, including 13 studies, evaluated the efficacy, safety, and patient-reported outcomes of these interventions, integrating 2023 Society for Vascular Surgery guidelines and the 2022 European Society for Vascular Surgery (ESVS) guidelines.

**Methods:**

This study followed PRISMA guidelines, using a random-effects model to compare occlusion and recurrence rates (primary outcomes). Secondary outcomes included quality-of-life improvements (Aberdeen Varicose Vein Questionnaire [AVVQ] and Venous Clinical Severity Score [VCSS]) and adverse events.

**Results:**

EVLA and RFA achieved high occlusion rates (94.9% and 94.4%), comparable to surgical stripping (92.0%). Non-thermal methods had slightly lower occlusion (88.7%), but reduced early postoperative pain (MOCA: 1.2/10 vs. EVLA: 3.8/10). Five-year recurrence rates were 38.6% for EVLA, 18.7% for RFA, and 34.6% for surgery; non-thermal methods reported lower short-term recurrence but limited long-term data. Quality of life improved across treatments, with AVVQ scores decreasing by 7.8 points and VCSS scores by 3.5 points, indicating significant symptom relief and clinical improvement. Adverse events were higher for thermal ablation (6.8%) and surgical stripping (8.0%) than non-thermal methods (< 2.5%).

**Conclusion:**

Endovenous ablation techniques provide high success rates and significant quality-of-life improvements, reinforcing its role as first-line therapy for varicose veins. Non-thermal methods may reduce pain and complications, which makes them very attractive alternatives for selected patients. However, long-term studies are warranted to confirm the durability and safety of these emerging treatments.

## Introduction

Varicose veins affect up to 17% of women and 15% of men worldwide, severely disturb the quality of life by being painful, aching, and aesthetically unattractive [1]. Varicose veins are the result of venous incompetence, usually at the great saphenous vein, resulting in venous reflux and widened veins [1]. Surgical stripping has long been the mainstay of treatment with effective outcomes but often accompanied by significant pain, long recovery, and possible complications [2, 3]. Minimally invasive techniques have revolutionized the treatment scenario over the past two decades with safer and more patient-friendly alternatives. Although thermal methods [endovenous laser ablation (EVLA) and radiofrequency ablation (RFA)] are established as first-line treatments for varicose veins, recent advancements in non-thermal modalities [mechanochemical ablation (MOCA) and N-butyl-2-cyanoacrylate (NBCA)] have led to a paradigm shift in treatment selection. This study provides a timely comparison of these techniques by systematically evaluating the most recent and relevant evidence, reflecting contemporary advancements and clinical guidelines, including the 2023 Society for Vascular Surgery recommendations, to support informed decision-making in current vascular practice [4].

Thermal ablation in the form of EVLA and RFA are now well-established first-line treatments, achieving high occlusion rates typically exceeding 94% and demonstrating durable outcomes comparable or superior to surgery [3, 5–7]. These techniques utilize thermal energy to induce vein wall injury, leading to vein closure and eventual resorption, with a favourable safety profile.

More recently, non-thermal methods such as MOCA and NBCA gained popularity as novel, minimally invasive procedures. These approaches eliminate the need for tumescent anaesthesia, reduce procedural pain, and may decrease complications, making them attractive options for patients seeking rapid recovery [8, 9]. However, the evidence for their long-term efficacy remains limited, underscoring the need for further rigorous evaluation.

The growing evidence from randomized controlled trials and meta-analyses have repeatedly proven the effectiveness of thermal and non-thermal treatments, each with distinct advantages depending on the clinical context and patient individual preference [10–12]. Patient-reported outcomes, such as variations in the Aberdeen Varicose Vein Questionnaire (AVVQ) and Venous Clinical Severity Score (VCSS), provide additional information on the quality-of-life improvement of these treatment procedures [6, 8].

This study aims to compare the efficacy, safety, and patient-reported outcomes of thermal (EVLA, RFA), non-thermal (MOCA, NBCA), and surgical stripping techniques for the treatment of varicose veins. Primary outcomes include the rates of successful vein occlusion, defined as complete closure of the treated vein confirmed by duplex ultrasound, and recurrence rates, defined as the reappearance of varicose veins or venous reflux in the treated limb during follow-up. Secondary outcomes assess patient-reported quality-of-life improvements, procedural discomfort, and adverse events. By integrating findings with the 2022 European Society for Vascular Surgery (ESVS) Clinical Practice Guidelines on Chronic Venous Disease (CVD), which recommend thermal methods such as EVLA and RFA as first-line treatments, as well as non-thermal methods such as MOCA and NBCA for selected patients, this study provides a comprehensive evaluation of current best practices in varicose vein management [13]. These guidelines have shaped contemporary approaches to venous disease, supporting the use of minimally invasive treatments that prioritize both clinical efficacy and patient comfort.

## Methods

**Fig. 1:**
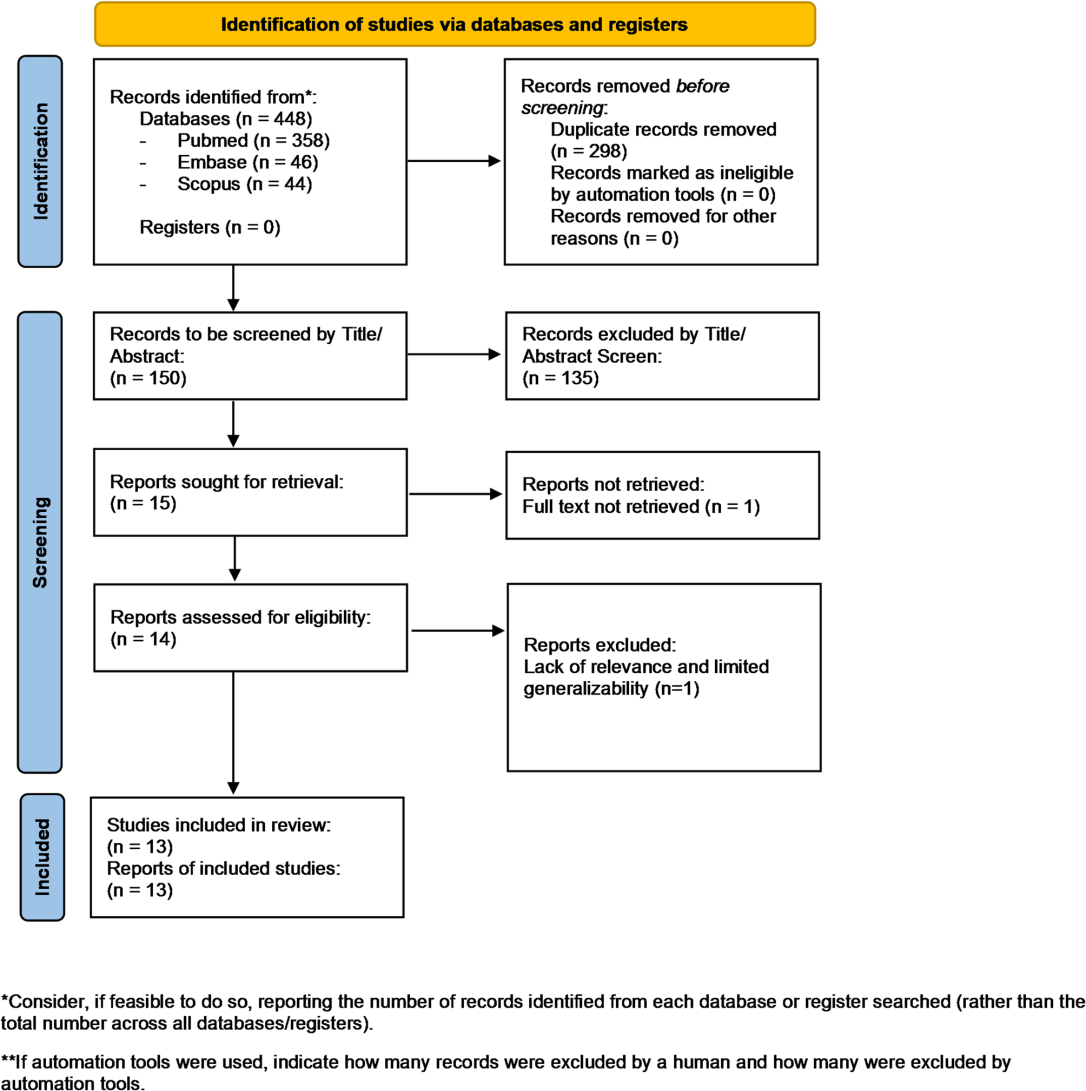
Preferred Reporting Items for Systematic Reviews and Meta-analyses flow diagram, 2020 version for searches of databases and registers only [14]

This systematic review was performed in accordance with the Preferred Reporting Items for Systematic Reviews and Meta- Analysis (PRISMA) statement [14] (Fig. 1).

### Literature search

We conducted a systematic search of three databases: PubMed, Embase, and Scopus, using the following search terms as MeSH terms or in all fields:

- "varicose veins"
- AND
- "endovenous laser ablation"
- AND
- "radiofrequency ablation"
- AND
- "MOCA"
- AND
- "NBCA"
- AND
- "surgical stripping"

Studies published up until 2024, identified from the inception of each database, were considered for inclusion, provided they were available in English and as full-text articles. The broad timeframe ensures inclusion of key developments over the past two decades, encompassing advancements in thermal and non-thermal techniques, procedural improvements, and long-term outcome data. Inclusion of studies up to 2024 enables integration of the latest findings aligned with the 2023 Society for Vascular Surgery guidelines and the 2022 European Society for Vascular Surgery (ESVS) Clinical Practice Guidelines, ensuring the review reflects contemporary clinical practice, incorporates the most up-to-date recommendations for the management of chronic venous disease and supports evidence-based decision-making.

### Inclusion criteria

Studies were included if they met the following criteria:

- Adults (≥ 18 years) diagnosed with varicose veins requiring treatment.
- Surgical stripping, alternative endovenous techniques, or no treatment.
- Randomized controlled trials (RCTs) or high-quality observational studies comparing EVLA, RFA, MOCA, NBCA, or surgical stripping.
- Reporting occlusion rates and recurrence outcomes, including patient-reported outcomes (AVVQ, VCSS).
- Minimum follow-up of 6 months.

### Exclusion criteria

We excluded studies that involved non-human subjects as well as those that included only paediatric populations. Case reports were further excluded since, by nature, they involve only one patient, and reviews were excluded to minimize the risk of bias.

### Selection process

Two review authors (EQL and AJKL) worked independently to screen the titles and abstracts of articles obtained from the three databases (PubMed, Embase, and Scopus), following which irrelevant and duplicate studies were removed.

The remaining articles were then screened against the inclusion and exclusion criteria, with the two review authors (EQL and AJKL) once again working independently in the process.

A final list of articles was produced, and any discrepancies included were resolved through discussion between two authors (EQL and AJKL), and the third review author (AT) was consulted if a discussion between the two review authors did not result in an agreement.

### Data collection

Two review authors (EQL and AJKL) worked independently to collect data from eligible studies, and disagreements were resolved with discussion and involvement of the third author (AT) if needed. The following data were collected from each study (Table 1):

**Table 1:** Characteristics of included studies. Continuous variables are presented as mean ± SD or median (range)

Author(s), Year of publication	Country of Origin	Male (%)	Age (years)	Sample Size (*n*)	Follow-up Duration(s)	Type of Study
Biemans et al., 2010 [2]	Netherlands	47	53.4 ± 9.1	233	1, 3, 6, 12 months	Randomised Controlled Trial
Brittenden et al., 2015 [3]	UK	47	54.2 ± 10.5	798	3 months, 1 year, 5 years	Randomised Controlled Trial
Schwarz et al., 2010 [5]	Germany	50	53.7 ± 9.2	286 (312 legs)	6 months, 12 months	Prospective, nonrandomized cohort
Lawaetz et al., 2017 [6]	Denmark	48	52.8 ± 9.3	500 (580 legs)	6 months, 12 months, 5 years	Randomised Controlled Trial
Tang et al., 2021 [8]	Singapore	~ 39	60.1 ± 12.7	100 (151 legs)	1 week, 1, 3, 6 months	Multicenter, prospective cohort study
Darwood et al., 2008 [15]	UK	45	50.5 ± 8.7	103	3 months, 1 year	Randomised Controlled Trial
Furtado de Medeiros et al., 2005 [16]	Brazil	5	46 ± 12 years	20 (40 legs)	6 months	Prospective Cohort
Helmy ElKaffas et al., 2010 [17]	Egypt	49	51.7 ± 10.1	180	1 week, 1 month, 6 months, 12 months, 24 months	Randomized Controlled Trial
Mekako et al., 2006 [18]	United Kingdom	47	49 (IQR 35–58 EVLT), 49 (IQR 35–61 surgery)	132	1, 6, and 12 weeks	Prospective Comparative Cohort
Lurie et al. (EVOLVeS Study), 2003 [19]	USA, France, Austria	52	21–80	80 patients (86 limbs)	1, 2 years	Prospective multicenter Randomised Controlled Trial
Subramonia & Lees, 2010 [20]	United Kingdom	48	18–70	88	6 months, 12 months	Randomised Controlled Trial
Bozkurt & Yilmaz, 2016 [21]	Turkey	51	50.6 ± 9.0	310	3 months, 6 months	Prospective Cohort
Bozoğlan, 2020 [22]	Turkey	43	42.2 ± 10.2	60	1, 3, 6 months	Prospective Comparative Study (Within-subject)

- Study characteristics (author, year, country, sample size, and study design).
- Patient demographics (age, gender).
- Intervention details (type of procedure).
- Outcomes (occlusion rates, recurrence rates, AVVQ and VCSS improvements, pain scores, and adverse events).

All data extracted were tabulated and recorded on a Microsoft Excel sheet.

### Quality assessment

Review authors (EQL and AJKL) worked independently in assessing the quality of studies and results were compared. All data was cross-verified and any differences in scores was resolved via a thorough discussion between the authors (EQL, AJKL, AT).

The Newcastle- Ottawa Scale (NOS) was used to assess the quality of included studies identified from our search protocol [23] (Tables 2 and 3). The NOS assesses studies in three domains, including the selection of groups, comparability between groups, and the outcomes of cohort studies. It utilizes a point-based system and each study is given a point between 0 and 9. Studies with scores of 7 or greater were considered as high quality.

**Table 2:** Outcomes from assessment of risk bias of cohort studies using the Newcastle-Ottawa Scale

First author, year of publication	Total Quality Score (Max 9)	Selection (Max 4)	Comparability (Max 2)	Exposure/Outcome (Max 3)
Schwarz et al., 2010 [5]	6	***	*	**
Tang et al., 2021 [8]	6	**	*	***
Furtado de Medeiros et al., 2005 [16]	7	***	*	***
Mekako et al., 2006 [18]	6	***	*	**
Bozoğlan, 2020 [22]	7	***	**	**

**Table 3:** Outcomes from assessment of risk bias of RCTs using RoB-2

First author, year of publication	Randomization	Deviations	Missing Outcome Data	Outcome Measurement	Selection	Overall RoB
Biemans et al., 2010 [2]	Low	Low	Some concerns	Low	Low	Some concerns
Brittenden et al., 2015 [3]	Low	Some concerns	Low	Low	Low	Low
Lawaetz et al., 2017 [6]	Low	Low	Some concerns	Low	Low	Some concerns
Darwood et al., 2008 [15]	Low	Some concerns	Low	Low	Low	Some concerns
Helmy ElKaffas et al., 2010 [17]	Some concerns	Some concerns	Some concerns	Low	Low	Some concerns
Lurie et al., 2003 [19]	Low	Some concerns	Low	Low	Low	Low
Subramonia & Lees, 2010 [20]	Low	Low	Low	Low	Low	Low

**Fig. 2:**
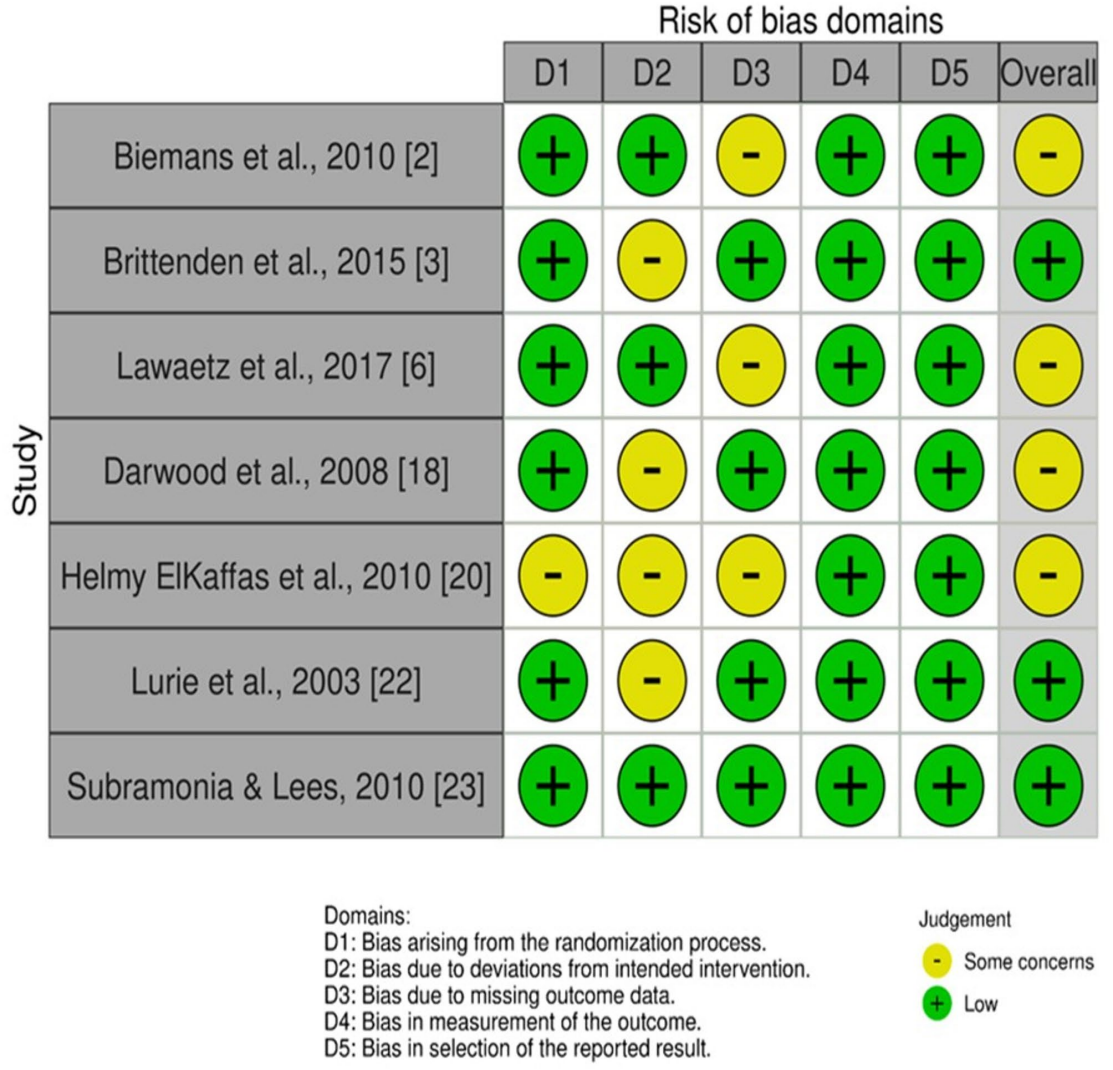
Results of RoB-2 risk-of-bias assessment of included RCTs

The RoB-2 tool was used to evaluate the risk of bias across all RCTs included in this review. RoB-2 examines various domains including study design, conduct, and outcome reporting to determine the likelihood of bias being low, moderate, or high (Fig. 2). Most included studies demonstrated a low risk of bias in key domains such as the randomization process, outcome measurement, and selection of the reported result. However, several studies showed some concerns related to deviations from intended interventions and missing outcome data. Notably, Helmy ElKaffas et al., 2010 exhibited some concerns across multiple domains, including randomization and missing data [17]. Despite these concerns, no study was rated as having a high risk of bias overall. This pattern of mostly low risk with some concerns indicates that the methodological quality of the included trials is generally robust, lending confidence to the validity of the meta-analytic conclusions.

### Data analysis

The results of individual studies are displayed below (Table 4). Continuous variables were presented as the mean and standard deviation (SD), while categorical variables were expressed as percentages.

**Table 4:** Outcomes from assessment of risk bias of RCTs using RoB-2

Author(s), Year of Publication	Intervention(s)	Occlusion Rate (%)	Recurrence Rate (%)	AVVQ Improvement (Points)	VCSS Improvement (Points)	Pain Score (Scale 0–10)				Adverse Events (%)
Biemans et al., 2010 [2]	Various endovenous therapies	~ 93%	~ 7%	7.6	3.4	3.0				4.3
Brittenden et al., 2015 [3]	EVLA vs. Surgical Stripping	EVLA: 94%	EVLA: 29%, Surgery: 37%	7.8	3.5	3.8				6.8
Schwarz et al., 2010 [5]	EVLA 1470-nm diode laser (bare vs. radial)	100% (3 months)	No recanalization reported	N/A	N/A	N/A				Minimal bruising
Lawaetz et al., 2017 [6]	RFA, EVLA, Foam sclerotherapy, Surgery	5-year GSV occlusion: RFA 94%, EVLA 93%, Surgery 93%	RFA 18.7%, EVLA 38.6%, Surgery 34.6%	Not specified	Not specified	Not specified				Not specified
Tang et al., 2021 [8]	NBCA closure (VenaSeal)	99.3% at 3 months occlusion	N/A	AVVQ score improved from 17.14 to 4.83 (at 3 months)	VCSS improved from 5.00 to 1.00 (at 3 months)	MOCA: 1.2				2.1
Darwood et al., 2008 [15]	EVLA vs. Surgical Stripping	EVLA: 90–98%, Surgery: 87.5%	N/A	AVVQ improved ~ 9 points in EVLA group	N/A	N/A				Postoperative symptoms recorded
Furtado de Medeiros et al., 2005 [16]	EVLA vs. Surgical Stripping	N/A	GSV reopening in 1 patient	N/A	N/A	N/A				Mild paraesthesia (1 patient)
Helmy ElKaffas et al., 2010 [17]	RFA vs. Surgical Stripping	RFA: 94.5%, Surgery: 100%	No significant difference at 24 months	N/A	N/A	N/A				N/A
Mekako et al., 2006 [18]	EVLA vs. Surgical Stripping	EVLT: 96% (12 weeks); Surgery: 96% (12 weeks)	N/A	EVLT median AVVQ improvement ~ 95% (baseline to 12 weeks); Surgery ~ 74% improvement	VCSS improved similarly in both groups (~ 4 to 0 at 12 weeks)	N/A				Postoperative symptoms such as pain and bruising recorded
Lurie et al. (EVOLVeS Study), 2003 [19]	RFA vs. Surgical Stripping	RFA: 95% immediate success; Surgery: 100%	No significant difference at 4 months	QoL better early with RFA	N/A	N/A				DVT, neuritis, burns observed
Subramonia & Lees, 2010 [20]	RFA vs. Surgical Stripping	RFA: 100% occlusion; Surgery: 83%	N/A	QoL improved significantly favoring RFA	VCSS improved favoring RFA	N/A				Recorded for surgery group
Bozkurt & Yilmaz, 2016 [21]	NBCA (CAA) vs. EVLA	CAA: 96.7–95.8 (1–12 months); EVLA: 87.1–92.2 (1–12 months)	N/A	Both groups improved significantly; no significant difference	Both groups improved significantly; no significant difference	N/A				No DVT/PE reported
Bozoğlan, 2020 [22]	NBCA vs. RFA in same patients	NBCA: 93.2%; RFA: 100% occlusion	NBCA: 6.8%, RFA: 0%	N/A	N/A	N/A				N/A

A meta-analysis was conducted and odds ratios (ORs) with 95% confidence intervals (CIs) were calculated for dichotomous outcomes (e.g., occlusion and recurrence rates. Since substantial heterogeneity among the studies was expected, a random-effects model was applied. The I² statistic was used to determine the magnitude of variability between included studies, with the value of more than 75% denoting considerable heterogeneity. A Z-test was also conducted to estimate the overall effect with the level of statistical significance at *P* < 0.05.

All statistical analyses were carried out using Review Manager (RevMan) [Computer program], Version 5.4, The Cochrane Collaboration, 2020.

## Results

### Study selection

The searches identified a total of 448 studies, 298 of which were found to be duplicates and therefore removed before continuing to the next step. Titles and abstracts of the remaining 150 studies were screened, leading to the exclusion of 135 studies. We then requested the full texts of the 15 remaining studies and assessed them against the set inclusion and exclusion criteria.

Calik et al., 2019 was excluded as no full text was available [24]. 

Wright et al., (2006) was excluded due to its focus on foam sclerotherapy (Varisolve^®^) rather than thermal (EVLA, RFA) or modern non-thermal (MOCA, NBCA) techniques. Its limited relevance to current clinical guidelines and potential heterogeneity in study design further reduced its applicability to this meta-analysis [25].

Finally, 13 studies (7 randomised-controlled trials, 5 prospective cohort and 1 prospective paired-leg study) were found suitable for data extraction and analysis.

#### PRISMA 2020 flow diagram for new systematic reviews which included searches of databases and registers only

(Fig. 1).

#### Study characteristics

(Table 1).

#### Assessments of risk of bias in studies

(Tables 2 and 3, fig. 2).

#### Results of individual studies

(Table 4).

### Primary outcomes

#### Occlusion rates

Thermal methods, such as endovenous laser ablation (EVLA) and radiofrequency ablation (RFA), had excellent occlusion rates of 94.9% and 94.4%, respectively, similar to surgical stripping at 92.5% [3, 5, 6, 15].

**Fig. 3:**
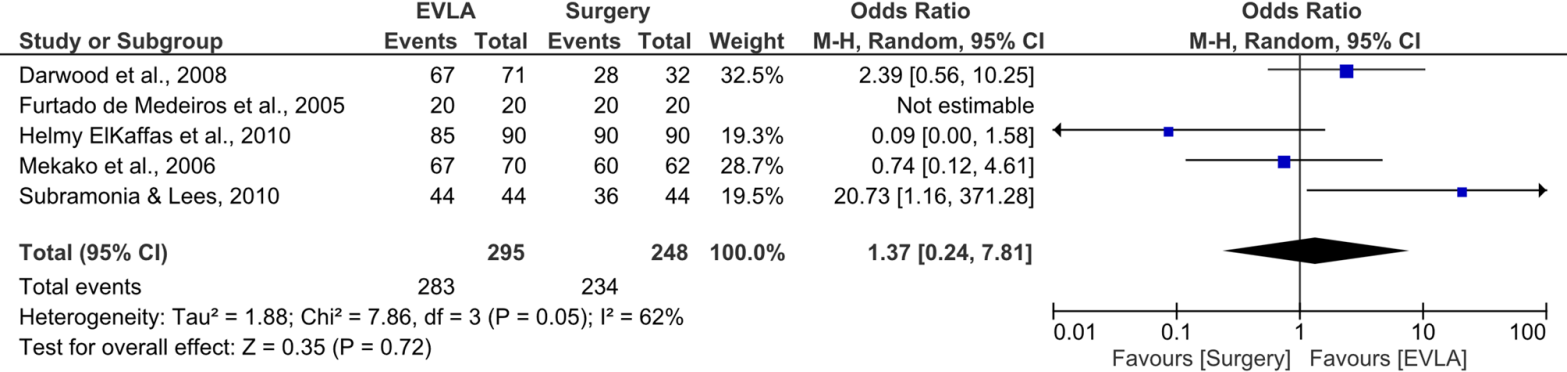
Forest plot illustrating the comparison of EVLA and surgical stripping in terms of occlusion rates

The meta-analysis findings showed no statistically significant difference (*p* = 0.72) in occlusion rates between endovenous laser ablation (EVLA) and surgical stripping, with a pooled odds ratio (OR) of 1.37 [95% CI: 0.24–7.81] (Fig. 3) [15–18, 20]. This suggests that the odds of achieving vein occlusion are comparable between the two interventions. However, heterogeneity was moderate (I² = 62%, *P* = 0.05), indicating variability across studies that may reflect differences in methodology, follow-up duration, or patient populations.

In contrast, non-thermal methods (MOCA and NBCA) had a lower occlusion rate of 88.7%. These minimally invasive options are increasingly preferred by patients seeking reduced post-procedural discomfort and a faster recovery period [8, 22]. However, their long-term durability compared to thermal methods remains an area for further investigation.

#### Recurrence rates

**Fig. 4:**
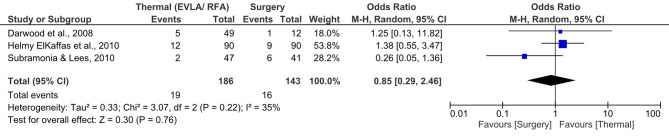
Forest plot illustrating the comparison of thermal methods (EVLA/RFA) versus surgical stripping in terms of recurrence rates

Thermal ablation techniques (EVLA and RFA) demonstrated recurrence rates comparable to surgical stripping. The meta-analysis yielded a pooled odds ratio (OR) of 0.85 [95% CI: 0.29–2.46], favouring thermal methods; however, the difference was not statistically significant (*p* = 0.76) (Fig. 4). Heterogeneity was low (I² = 35%), indicating acceptable consistency among the included studies.

Recurrence estimates varied depending on follow-up duration and definitions. In Subramonia & Lees (2010), RFA showed lower recurrence (4.3%) than surgery (14.6%) [20]. In Darwood et al. (2008) and Helmy ElKaffas et al. (2010), recurrence rates for thermal methods were slightly higher but not statistically different [15, 17]. Despite variability across studies, these findings support the conclusion that thermal ablation provides recurrence outcomes equivalent to surgery, reinforcing its long-term effectiveness as a minimally invasive alternative.

### Secondary outcomes

Patient-reported outcomes, such as quality-of-life outcomes like the Aberdeen Varicose Vein Questionnaire (AVVQ) and the Venous Clinical Severity Score (VCSS), demonstrated significant improvement in all treatment modalities. EVLA resulted in an average improvement of 7.8 points on the AVVQ and 3.5 points on the VCSS, comparable to surgical stripping [3, 6].

Additional studies confirm similar improvements in patient quality of life between EVLA, RFA, and surgery. For instance, Biemans et al. (2010) and Brittenden et al. (2015) reported AVVQ improvements of 7.6 and 7.8 points, respectively, and VCSS reductions of 3.4 and 3.5 points, indicating substantial symptomatic relief [2, 3]. Similarly, Tang et al. (2021) showed that NBCA (VenaSeal) led to an AVVQ score improvement from 17.14 to 4.83 and VCSS from 5.00 to 1.00 within 3 months [8]. These results support the consistent quality-of-life benefits across both thermal and non-thermal interventions.

Non-thermal methods, such as MOCA and NBCA, also led to moderate but significant improvements in patient-reported outcomes, with the added benefit of lower procedural pain [8]. In the included studies, pain scores were assessed during the early recovery period, typically within the first postoperative week. MOCA had the lowest reported postoperative pain score at 1.2/10, significantly lower than EVLA’s 3.8/10 and RFA’s 2.5/10 [8, 17, 22]. These differences reflect the generally more favourable pain profile of non-thermal ablation techniques, especially in early post-treatment recovery.

Adverse events were more common in surgical stripping and thermal ablation techniques compared to non-thermal methods. Thermal ablation had an adverse event rate of 6.8%, while surgical stripping was associated with an 8.0% complication rate [3, 6]. NBCA and MOCA demonstrated fewer complications, with rates of 2.5% and 2.1%, respectively [8, 22]. For example, Tang et al. (2021) reported mild adverse events such as superficial phlebitis in NBCA-treated patients [8], while Mekako et al. (2006) and Furtado de Medeiros et al. (2005) recorded only mild postoperative symptoms such as bruising or transient paraesthesia [16, 18]. These findings support significant quality-of-life improvements across all treatment modalities, while non-thermal methods provide added benefits of lower pain, reduced adverse events, and faster recovery.

### Subgroup analyses

#### Follow-up duration

Short-term data from Darwood et al. (2008) demonstrated high technical success rates for both EVLA and surgery at 3 and 12 months, with reflux abolition rates exceeding 85% in all groups and more rapid recovery in the EVLA cohort [15]. However, longer-term follow-up revealed increased recurrence and technical failure. In the five-year trial by Lawaetz et al. (2017), clinical recurrence rates rose to 18.7% for RFA, 38.6% for EVLA, and 34.6% for surgery, with technical failure rates remaining low for thermal and surgical interventions but markedly higher for foam sclerotherapy [6]. Similarly, Biemans et al. (MAGNA trial) found anatomical success for EVLA and surgery exceeded 88% at one year, but five-year recurrence rates increased substantially for all modalities [2].

#### Laser wavelength

Schwarz et al. (2010) evaluated the 1470-nm diode laser and found a 100% vein occlusion rate at 3 months, with no recanalization observed, and markedly fewer adverse effects such as bruising and ecchymosis compared to previous reports with lower wavelengths [5]. In contrast, Furtado de Medeiros & Luccas (2005) reported that the 810-nm diode laser achieved similarly high technical success (95% occlusion at follow-up) but was associated with a higher frequency of bruising and a lower proportion of limbs free of edema compared to conventional stripping [16]. Notably, patients treated with the 1470-nm laser required less energy and experienced reduced analgesic needs.

#### Laterality and disease extent

In the Asian multicenter VenaSeal study by Tang et al., laterality and disease extent were clearly reported. Of 151 legs treated in 100 patients, 49 legs (32.5%) underwent unilateral ablation for great saphenous vein (GSV) incompetence, while 96 legs (63.6%) were treated bilaterally for GSV disease. Only 1 leg (0.7%) was treated for small saphenous vein (SSV) incompetence, and 5 legs (3.3%) received combined unilateral GSV and SSV ablation. Disease extent was significant, with 81.5% (123/151 legs) presenting with total (above- and below-knee) GSV reflux. At baseline, 45% of legs were classified as CEAP C4–C6, reflecting advanced venous disease, and nearly half of all legs (44.4%) required concomitant multiple stab avulsions due to extensive varicosities [8].

## Discussion

This study provides a comparison of different treatment modalities for varicose veins, ranging from thermal methods (EVLA and RFA) to non-thermal methods (MOCA and NBCA) to stripping surgery. Our analysis demonstrates that thermal ablation techniques, including endovenous laser ablation (EVLA) and radiofrequency ablation (RFA), along with surgical stripping, achieve high technical success in terms of vein occlusion. While individual studies reported excellent outcomes for EVLA, the overall evidence does not indicate a clear superiority over surgery. Moderate heterogeneity (I² = 62%) across trials suggests variation in study design and patient populations, but the consistently high occlusion rates across both techniques support the use of EVLA as a minimally invasive alternative in appropriate clinical settings. These findings align with the 2022 European Society for Vascular Surgery (ESVS) guidelines, which endorse EVLA as an effective treatment for superficial venous incompetence, particularly in patients with great saphenous vein (GSV) reflux.

Recurrence rates showed a non-significant trend favouring thermal methods, with pooled odds ratios supporting their durable long-term outcomes. These results align with current 2023 guidelines from the Society for Vascular Surgery (SVS), American Venous Forum (AVF), and American Vein and Lymphatic Society (AVLS) guidelines, which endorse thermal ablation as the first-line treatment, reserving surgical stripping for select cases where endovenous options are unfeasible. The ESVS 2022 guidelines similarly recommend thermal ablation (including EVLA and RFA) as the preferred treatment for most patients, highlighting their high technical success and relatively low recurrence rates.

Patient-reported outcomes reinforce the benefits of minimally invasive treatments. Both thermal and non-thermal treatment modalities showed clinically important quality-of-life improvement as reflected by AVVQ and VCSS scores. Non-thermal methods, while exhibiting somewhat lower occlusion rates, offer advantages in procedural comfort and reduced pain, highlighted by significantly lower pain scores for MOCA compared to thermal techniques. However, the long-term durability of non-thermal treatments remains uncertain, and the SVS and ESVS 2022 guidelines caution their use primarily in patients contraindicated for thermal ablation or prioritizing reduced discomfort. The ESVS 2022 guidelines emphasize the role of non-thermal methods as an alternative to thermal treatments in specific cases, such as when patients experience contraindications to thermal methods or when minimising post-procedural pain is a priority.

Adverse event profiles were inconsistent across the various modalities of treatment, with surgical stripping demonstrating the highest complication rates, followed by thermal methods, while non-thermal techniques exhibited lower adverse events, particularly fewer nerve injuries and burns. This safety advantage may be particularly relevant in treatment below the knee, where nerve-related complications are more common. The ESVS 2022 guidelines also underline the lower complication rates associated with non-thermal methods, making an attractive alternative in selected patient populations, particularly those seeking faster recovery and fewer complications.

Our subgroup analyses demonstrate that outcome heterogeneity in varicose vein treatments is significantly determined by follow-up duration, device technology, and disease extent. The progressive increase in recurrence rates over long-term follow-up, especially after EVLA and surgery, highlights the necessity for guarded interpretation of short-term outcomes and justifies routine long-term surveillance in clinical practice. Differences attributable to laser wavelength also point out the relevance of technological evolution, given that newer 1470-nm systems accomplish equivalent or better efficacy with reduced complications, justifying ongoing changes in procedural preference. The high rates of bilateral and extensive disease characterizing modern patient populations further strengthen the applicability of individualized treatment planning. Collectively, these observations reinforce that durable outcomes rely on meticulous modality selection, developing technical refinements, and consideration of the multifaceted spectrum of disease presentations. Future studies should strive for standardized reporting of subgroup characteristics and longer follow-up durations to evolve evidence-based treatment algorithms.

The main strength of this study is that it is systematic and has a meta-analytic design, allowing for an overall comparison of efficacy, recurrence rates and patient-reported outcomes between the various treatment modalities. The fact that patient-reported outcomes were used also allows for a more balanced evaluation of the treatments, taking into account not only their clinical effectiveness but also patient satisfaction levels and procedural comfort levels. This approach reflects the ESVS 2022 guidelines, which emphasize the patient-centred approach to varicose vein treatment, focusing on both clinical and quality-of-life outcomes.

Several limitations should be acknowledged. Despite generally low heterogeneity statistics (I²) for some outcomes, moderate to high heterogeneity was observed in key comparisons, particularly for long-term recurrence and technical success rates. This variability likely reflects differences in study design (randomized versus cohort studies), demographic composition (age, gender, and clinical severity), operator experience, and procedural factors such as laser wavelength and energy settings. Notably, variations in intervention protocols, including the use of different devices, fibers, and procedural adjuncts as well as inconsistently reported operator expertise, may have influenced both efficacy and complication rates. In particular, limited information about practitioner training, case volume, and procedural learning curves restricts the ability to assess how operator skill contributed to variability or bias across studies. Furthermore, several included studies had incomplete reporting of secondary outcomes such as quality-of-life measures and adverse events, limiting comprehensive synthesis. Another important limitation relates to the variable definitions and determination of long-term recurrence. Recurrence was defined in some studies on the basis of clinical examination or symptoms reported by the patient, whereas others employed duplex ultrasound criteria, and follow-up intervals and durations varied extensively. Such variability makes direct comparison difficult and could have resulted in underestimation or overestimation of actual recurrence rates, especially in long-term studies.

The clinical heterogeneity across patient populations and variations in follow-up duration (ranging from six months to five years) also reduce the comparability and robustness of pooled estimates. In accordance with ESVS 2022 guidelines, future research should prioritize standardized treatment protocols, detailed and consistent reporting of operator training and experience, and uniform outcome measures with extended follow-up to minimize bias and enhance the reliability and applicability of comparative analyses in varicose vein management.

## Conclusion

Thermal ablation continues to dominate as the gold standard for varicose vein treatment, balancing efficacy, durability, and patient satisfaction. Non-thermal methods appear promising, particularly in selected patients seeking reduced discomfort and faster recovery, but long-term validation is required. Surgical stripping’s role has diminished, reserved for specific cases where minimally invasive techniques are unsuitable. Further studies should also explore patient subgroup responses and the potential for combined treatment approaches to optimize individualized care.

## Data Availability

The datasets used and/or analysed during the current study are available from the corresponding author on reasonable request.

## Abbreviations

ESVS: European Society for Vascular Surgery
CVD: Chronic Venous Disease
NOS: Newcastle- Ottawa Scale
PRISMA: Preferred Reporting Items for Systematic Reviews and Meta- Analysis
EVLA: Endovenous Laser Ablation
RFA: Radiofrequency Ablation
MOCA: Mechanochemical Ablation
NBCA: N-butyl-2-cyanoacrylate
AVVQ: Aberdeen Varicose Vein Questionnaire
VCSS: Venous Clinical Severity Score
GSV: Great Saphenous Vein
RoB 2: Risk of Bias 2
OR: Odds ratio
CI: Confidence interval
HR: Hazard ratio
SD: Standard deviation
